# Exosomal circRNA as a novel potential therapeutic target for multiple myeloma-related myocardial damage

**DOI:** 10.1186/s12935-021-02011-w

**Published:** 2021-06-13

**Authors:** Runjie Sun, Wei Liu, Yangang Zhao, Haoyu Chen, Zhenzhen Wang, Yanyu Zhang, Xiaoqi Sun, Xing Cui

**Affiliations:** 1 College of Traditional Chinese Medicine, Shandong University of Traditional Chinese Medicine, 16369 Jingshi Road, Jinan, 250014 China; 2 College of Nursing, Shandong University of Traditional Chinese Medicine, 16369 Jingshi Road, Jinan, 250014 China; 3 Department of Audit, Shandong University of Traditional Chinese Medicine, 16369 Jingshi Road, Jinan, 250014 China; 4 Department of Cardiology, Affiliated Hospital of Shandong University of Traditional Chinese Medicine, 16369 Jingshi Road, Jinan, 250014 China; 5 Department of Hematology, Affiliated Hospital of Shandong University of Traditional Chinese Medicine, 16369 Jingshi Road, Jinan, 250014 China

**Keywords:** Multiple myeloma, Myocardial damage, Exosome, Circular RNA

## Abstract

**Introduction:**

Myocardial damage is a mostly incurable complication of multiple myeloma (MM) that seriously affects the treatment outcome and quality of life of patients. Exosomal circular RNAs (exo-circRNAs) play an important role in tumor occurrence and development and are considered key factors in MM pathogenesis. However, the role and mechanism of action of exo-circRNAs in MM-related myocardial damage are still unclear. This study aimed to investigate correlations between exo-circRNAs and MM and to preliminarily explore the role of exo-circRNAs in MM-related myocardial damage.

**Methods:**

Six MM patients and five healthy controls (HCs) were included in the study. High-throughput sequencing and qRT-PCR verification were used to obtain a profile of abnormally expressed exo-circRNAs. GO, KEGG, miRanda, TargetScan and Metascape were used for bioinformatics analyses. H9C2 cells treated with exosomes from U266 cells were used in cell experiments. CCK-8, PCR, immunofluorescence and western blotting assays were used to detect cell proliferation and expression of autophagy-related indicators. Electron microscopy was used to observe the number of autophagic vesicles.

**Results:**

Bioinformatics analysis showed that circRNAs with upregulated expression had the potential to promote MM-related myocardial damage. In addition, PCR results confirmed that circ-G042080 was abundantly expressed in the serum exosomes of 20 MM patients. Correlation analysis showed that the expression level of circ-G042080 was positively correlated with the clinical level of MM and MM-related myocardial damage and that circ-G042080 might interfere with MM-related myocardial damage through a downstream miRNA/TLR4 axis. Cell experiments demonstrated that the circ-G042080/hsa-miR-4268/TLR4 axis might exist in H9C2 cells incubated with exosomes and cause abnormal autophagy.

**Conclusion:**

Abnormal expression of serum exo-circRNAs was found to be associated with MM-related myocardial damage, suggesting that exo-circRNAs might become a new diagnostic marker of MM-related myocardial damage and a therapeutic target.

**Supplementary Information:**

The online version contains supplementary material available at 10.1186/s12935-021-02011-w.

## Introduction

Multiple myeloma (MM) is the second most common hematological neoplastic disease after non-Hodgkin's lymphoma, accounting for 1% of all cancers and 10% of all hematological malignancies [1]. MM is characterized by the clonal proliferation of malignant plasma cells and the deposition of monoclonal immunoglobulin (M protein) in the bone marrow. M protein light chains and polysaccharide complexes are deposited in tissues and organs and can damage corresponding organ functions, leading to hypercalcemia, renal failure, anemia, bone lesions and cardiac comorbidities [2]. Among them, cardiac comorbidities are one of the most serious complications and can lead to cardiomyopathy and heart failure caused by cardiac amyloidosis and/or anemia. In addition, certain treatments used for MM can affect cardiac function [3]. Clinical data have confirmed that up to 50% of MM patients have heart damage [4]. In addition, cardiac comorbidities often have an insidious onset and lack specific clinical manifestations, and clinicians are prone to miss the diagnosis, thus delaying diagnosis and treatment. Therefore, we urgently need new targets for MM-related myocardial damage comorbidities to diagnose or treat the disease.

Circular RNAs (circRNAs) are a group of endogenous noncoding RNAs that are present in exosomes and plasma and are characterized by a covalent closed loop structure lacking a polyadenylated tail [5]. The latest research has revealed that circRNAs can act as microRNA (miRNA) sponges, protein translation templates and immunomodulators [6] and play a key role in regulating gene expression. Because circRNAs are closely related to disease development and are highly conserved and stable, they have become a hot spot in current research [7], especially their role as competitive endogenous RNA (ceRNA) and miRNA sponges. A large number of studies have confirmed that circRNAs can sequester miRNAs through complementary RNA base pairing and prevent miRNAs from binding to their mRNA targets [8], thus forming a circRNA-miRNA-mRNA network that interferes with disease occurrence and development. Studies have shown that the sponge mechanism of circRNAs is widely present in many diseases. For example, in gynecologic cancers [9, 10], liver cancer [11], bladder cancer [12], gastric cancer [13], breast cancer [14], glioma [15], hematological malignancies [16–18], and diabetes [19], among other diseases, circRNAs have been reported as promising tumor biomarkers and therapeutic targets. At present, upregulation or downregulation of circRNA expression has also been found in MM, and miRNAs have been indicated to be functionally deregulated or abnormally expressed in MM cells [20], suggesting that circRNAs can interfere with myeloma cell proliferation and MM development through a sponge mechanism [21, 22]. In addition, studies have found that circRNAs play an important role in regulation of cardiac function [23, 24]. Therefore, circRNAs have broad application prospects in MM and MM-related cardiac damage.

Exosomes are small extracellular vesicles (EVs) [25] with a diameter between 30 and 100 nm. In humans, exosomes are widely present in various body fluids, such as saliva, serum, plasma, urine and malignant tumor effusion. Exosomes express various characteristic surface markers, proteins and other substances and can transport circRNAs, mRNAs and other noncoding RNAs [26]. Exosomes transfer the molecules or biological information they carry to target cells through endocytosis or direct fusion with the target cell membrane [27], thereby mediating intercellular communication between the parental cell and neighboring cells and distant tissues. This effect is currently considered to be an important promoter of tumor disease progression [27]. In addition, exosomes can induce the activation, proliferation, differentiation and death of target cells. Therefore, they have high value in clinical research [28, 29]. MM disease progression largely depends on the bone marrow microenvironment, and communication mediated by EVs is an important aspect of this environment. Studies have found that exosomal miRNAs play an important role in MM progression and targeted therapy [20, 30–32]. Unfortunately, research on the role of exosomal circRNAs (exo-circRNAs) in MM and MM-related heart damage is still relatively rare. Therefore, we sequenced exo-circRNAs in the serum of MM patients, constructed a circRNA profile and combined these data with patient clinical symptoms to explore the relationship between exo-circRNAs and MM-related myocardial damage. In addition, this work provides a new potential exo-circRNA target for MM-related myocardial damage.

## Materials and methods

### Clinical serum specimens

Twenty MM patients who were admitted to the Department of Hematology, Affiliated Hospital of Shandong University of Traditional Chinese Medicine, were enrolled in the study. Five healthy adult volunteers were included in the experiment as healthy controls (HCs). The peripheral blood of the subjects was collected with a vacuum coagulation tube, and the serum was obtained after centrifugation (5 min, 2500 rpm) and stored at − 80 °C. The study was conducted in accordance with the Helsinki Declaration revised in 2008, and informed consent was obtained from all participants. All experimental methods were approved by the Ethics Committee of the Affiliated Hospital of Shandong University of Traditional Chinese Medicine.

### Extraction of exosomes

Exosomes were isolated using an ExoQuick exosome precipitation kit (System Bioscience, Palo Alto, CA, USA; EXOQ5A-1) according to the manufacturer’s instructions. Briefly, 250 µl of sample was mixed with 63 µl ExoQuick exosome precipitation solution, incubated at 4 °C for 30 min, and then centrifuged (30 min, 1500 × g). The supernatant was aspirated, the sample was centrifuged again (5 min, 1500 × g), and the obtained pellet was resuspended in 100 µl PBS and stored at − 80 °C.

### Morphological identification of exosomes

The exosomes were fixed with the same amount of 4% paraformaldehyde and washed with phosphate-buffered saline (PBS; pH = 7.4). Then, the samples were placed on formvar/carbon 200-mesh copper grids for 20 min at room temperature and fixed in 1% glutaraldehyde for 5 min. Next, the samples were stained with uranyl oxalate for 5 min, and 4% uranyl acetate and 2% methyl cellulose were added at a ratio of 1:9 on ice. Finally, the grids were imaged via transmission electron microscopy (TEM) (Hitachi, HT7700).

### Characterization of exosomes

The exosomes were diluted with PBS (1:1000) and analyzed with a NanoSight NS300 particle analyzer (NanoSight, Amesbury, UK). The concentration (particles/ml) and particle size (nanometer) of exosomes were determined via nanoparticle tracking analysis (NTA).

### Identification of exosome marker proteins

The exosomes were dissolved in RIPA lysis buffer (Sparkjade, Jinan, China, EA0002), and total protein was extracted. The protein concentration was determined using a bicinchoninic acid (BCA) protein detection kit (Beyotime, Shanghai, China, P0011). The protein was separated via sodium dodecyl sulphate–polyacrylamide gel electrophoresis (SDS-PAGE; Sparkjade, Jinan, China; EC0004) and transferred to a polyvinylidene fluoride (PVDF) membrane (Sparkjade, Jinan, China, ED0004). The PVDF membrane was soaked in 5% skimmed milk for 2 h and then incubated with anti-calnexin, anti-TSG101 and anti-CD63 antibodies overnight at 4 °C. After incubation with secondary antibody for 1 h, the images were analyzed using an Alpha Innotech Fluor Chem Q Imaging Analysis System. The relative TSG101 and CD63 protein levels were normalized to the optical density of calnexin.

### Western blotting

Cells were lysed in RIPA lysis buffer (Sparkjade,), and the protein concentration was measured with a BCA protein assay kit (Beyotime). Equal amounts of sample protein were separated in 10% SDS-PAGE gels and transferred onto PVDF membranes (Sparkjade). The samples were blocked with 5% skimmed milk at room temperature for 2 h and then incubated with anti-calnexin, anti-TSG101, anti-CD63 (Santa Cruz Biotechnology, Santa Cruz, CA, USA), anti-Toll-like receptor 4 (TLR4; Abcolonal; A5258), anti-LC3B (Abcolonal; A19665), anti-SQSTM1/p62 (Abcolonal; A19700) or anti-Beclin 1 (Abcolonal; A7353) antibody overnight at 4 °C. After washing with PBS 3 times, diluted secondary antibody was added and incubated with the membrane at room temperature for 1 h, and the membrane was washed 3 consecutive times with PBS. Finally, an Alpha Innotech Fluor Chem Q imaging analysis system was used to visualize the bands. The relative TLR4, LC3, Beclin 1 and P62 protein levels were normalized to the optical density of Actin-1.

### RNA extraction and real-time qPCR verification

Total RNA was extracted from exosomes using an RNA extraction kit (Sparkjade, TAKARA.9767), and the concentration and purity of extracted RNA were measured with a NanoDrop 2000 spectrophotometer (Thermo). RT-PCR and SYBR Green qPCR kits (AH0401) were used to verify the RNA (Sparkjade), and PCR was run on a Roche LightCycler 480 system. According to the procedures provided in the instructions, the circ-G024080 and hsa-mir-4268 expression levels, and the TLR4, LC3 and GAPDH mRNA levels were detected. Each sample was tested at least 3 times, and the changes in relative gene expression were calculated using the formula 2^−(ΔΔCt)^. GAPDH was used as an internal control, and the results for each sample were normalized to GAPDH expression (See Table 1). The primer sequences were as follows:

**Table 1:** Gene primer sequences

Gene	Forward	Reverse
circ-G024080	AGGGAGGAGGCTGTTAGAGG	CTTCCTCACTCCAGCCAGTG
hsa-mir-4268	GGCTCCTCCTCTCAGGAT	GGTCCAGTTTTTTTTTTTTTTTCCACA
TLR4	TCCAGAGCCGTTGGTGTATC	AGAAGATGTGCCTCCCCAGA
LC3	TTCCTCCTGGTGAATGGGCA	TTGCCTTGGTAGGGGCTTAAC
GAPDH	GGCCTCCAAGGAGTAAGACC	AGGGGAGATTCAGTGTGGTG

### Cell viability

H9C2 (XYXB-1190) and U266 (EY-X1023) cells were obtained from ATCC (Rockville, MD, USA). H9C2 cells were cultured in Dulbecco’s modified Eagle’s medium (DMEM) supplemented with 10% fetal bovine serum (FBS), and U266 cells were cultured in Roswell Park Memorial Institute (RPMI) 1640 medium supplemented with 10% FBS. The cells were used in subsequent experiments after reaching the logarithmic growth phase. U266 cell exosomes were extracted for the following experiment.

H9C2 cells were cultured in 96-well plates at 1 × 10^5^ cells per well. The cells were divided into three groups: a control group (H9C2 only), exosome group (H9C2 cells incubated with U266 exosomes) and GW4869 group (H9C2 cells incubated with U266 exosomes and then with GW4869). The cells in the three groups were incubated at 37 °C and 5% CO_2_ for 12 h. Subsequently, according to the manufacturer's instructions, a Cell Counting Kit-8 (CCK-8) assay (Beyotime, Shanghai, China, C0039) was used to determine cell viability. Each group was tested three times.

### Bioinformatics analysis

Briefly, after identification of the significantly differentially expressed circRNAs between the MM group and the HC group, Cluster and TreeView software were used to perform hierarchical cluster analysis. GO and KEGG analyses were used to evaluate the functions and pathways of differentially expressed genes in the MM and HC groups. TargetScan and miRanda were used to predict interactions between circRNAs and downstream miRNAs, and Cytoscape software (v2.8.0) was used to analyze and construct a ceRNA network.

### High-throughput sequencing

Total RNA was isolated from the samples, and the quality of the library was determined using a BioAnalyzer 2100 system (Agilent Technologies, Palo Alto, CA, USA). According to the supplier's instructions, ribosomal RNA (rRNA) was removed from the samples using an NEBNext rRNA Depletion Kit (New England Biolabs, Ipswich, Massachusetts, USA), and then, a sequencing library was constructed with an NEBNext® Ultra™ II Directional RNA Library Prep Kit (New England Biolabs). The BioAnalyzer 2100 system was used for library quality control and quantification, and an Illumina HiSeq instrument (Illumina) was used for 150-bp paired read sequencing.

### Luciferase reporter constructs and luciferase activity assay

Direct interactions between the partners in the circ-G042080-mediated ceRNA network were evaluated using a luciferase activity assay. A luciferase reporter vector (GP-mirGLO Dual-Luciferase miRNA Target Expression Vector; Promega) was used to produce luciferase constructs. circ-G042080 and the 3’UTR of TLR4 were cloned via RT-PCR. circ-G042080-WT, circ-G042080 mutant and TLR4-3’UTR (WT and mutant) were constructed. 293 T cells were seeded into 24-well plates and allowed to grow for 24 h without antibiotics before transfection. The cells were transfected with the constructed reporter vectors (300 ng) together with the miRNA (hsa-miR-4268) mimics or negative control mimics (100 nM) using Lipofectamine 2000 (2 μl). The cells were lysed ~ 24 h after transfection, and the luciferase activity was assayed using a Dual-Luciferase Reporter Assay System (Promega). Firefly luciferase activities were normalized to Renilla luciferase activities. All experiments were performed independently in triplicate.

### Cell counting kit-8 (CCK-8) assay

The experimental grouping was the same as described in Section “Cell viability”. After reaching the logarithmic growth phase, the cells were plated in a 96-well plate at 1 × 10^5^ cells/well and cultured for 2 h in a 37 °C incubator. Then, 10 µl of CCK-8 solution was added to each well and incubated with the cells for 1 h. The OD value was detected at 450 nm using a microplate reader to calculate the cell proliferation rate. Relative cell proliferation rate = (experimental OD-control OD)/control OD.

### Statistical analysis

The experimental data are presented as the mean ± standard deviation (SD). SPSS 26.0 software was used for statistical analysis. A t test was used for comparisons between two groups, and correlation analysis was used to evaluate the relationship between every two factors. Receiver operating characteristic (ROC) curves were used to determine the diagnostic sensitivity and specificity of disease-related indicators. Univariate and multivariate Cox regression models were used to determine factors affecting prognosis. Except for the univariate Cox regression model with P < 0.1 indicating statistical significance, in other statistical analysis methods, P < 0.05 indicated a significant difference.

## Results

### Identification and analysis of exosomes from MM patients

**Fig. 1:**
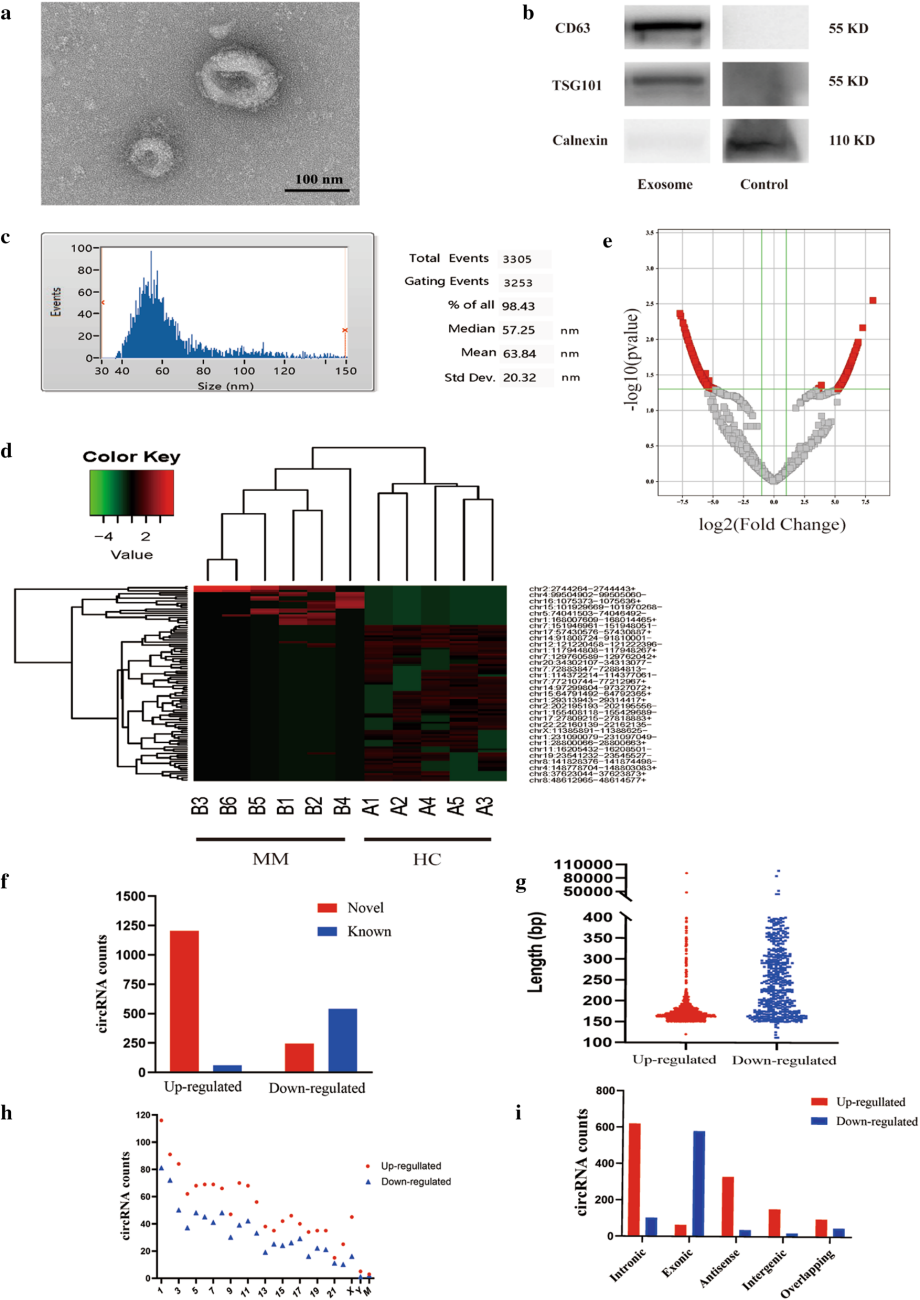
Detection of serum exosomes and analysis of exosomal circRNAs. **A** The size and morphology of serum exosomes detected by TEM. **B** CD63, TSG101 and calnexin protein expression was evaluated via WB. **C** The size and concentration of serum exosomes. **D** A total of 1265 upregulated circRNAs and 787 downregulated circRNAs were identified in MM patients compared with HCs. Red indicates upregulation, and green indicates downregulation. **E** Volcano plot showing the profile of differentially expressed exo-circRNAs in MM patients vs HCs. **F** Differential expression of exo-circRNAs in MM patients compared with HCs indicated by the volcano plot. **F** Among 2052 significantly differentially expressed circRNAs (fold change > 2, P value < 0.05), only 604 circRNAs were known, and 1448 circRNAs were novel. **G** Distribution of the 2052 circRNAs based on the predicted sequence length. **H** The 2052 circRNAs were distributed on 22 autosomes, sex chromosomes (X, Y) and mitochondrial chromosomes (**M**). **I** The circRNA categories to which each of the 2052 circRNAs belongs

In this study, 6 MM patients and 5 HCs were enrolled for high-throughput sequencing of serum exo-circRNAs. The serum exosomes were identified by TEM, western blotting (WB) and NTA. TEM showed that the exosomes were round and less than 120 nm in diameter. (Fig. 1A). Common protein expression markers, including CD9, CD63, CD81, and Tsg101, can be used to identify exosomes [33]. WB results demonstrated that what we collected from MM patients’ serum were exosomes (Fig. 1B). Furthermore, NTA results indicated that the average diameter of exosomes was 63.84 ± 20.32 nm (Fig. 1C). Together, the above results confirmed that our samples were exosomes. High-throughput sequencing showed that there were 10,106 differentially expressed circRNAs in the MM group compared with the HC group, of which 10,321 were upregulated and 5785 were downregulated (Fig. 1D). Next, fold change (FC) ≥ 2.0 and P ≤ 0.05 were used as the criteria for significant differences, and a total of 1265 upregulated circRNAs and 787 downregulated circRNAs were identified (Fig. 1E).

We analyzed and summarized the identified circRNAs based on origin, length, location and category. Among all the differentially expressed circRNAs, only 604 were known and were available in circBase and other databases (Fig. 1F). The median lengths of upregulated and downregulated circRNAs in the MM group were 168 nt and 345 nt, respectively (Fig. 1G), indicating that MM exosomes mainly carry small circRNAs less than 500 nt in length. The results also showed that the differentially expressed circRNAs were distributed in 22 autosomes, sex chromosomes and mitochondria in humans. No specific chromosome of origin was found (Fig. 1H). In addition, most of the significantly upregulated circRNAs in the MM group were sense overlapping circRNAs, while most of the downregulated circRNAs were exonic (Fig. 1I).

### Bioinformatics analysis of differentially expressed circRNAs

**Fig. 2:**
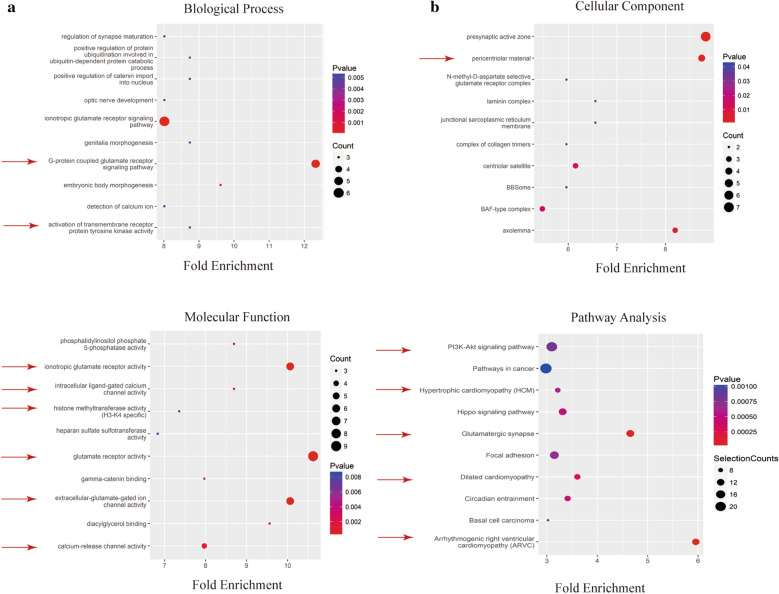
GO and KEGG analyses of significantly differentially expressed circRNAs. The top 10 enriched biological process terms (**A**), cellular component terms (**B**), and molecular function terms (**C**) determined by GO analysis. **D** The top 10 enriched signaling pathways based on KEGG analysis

GO and KEGG analyses were used to predict the functions and signaling pathways of exo-circRNAs in MM. Previous results showed that the number of upregulated circRNAs was significantly larger than that of downregulated circRNAs in the MM group. Therefore, we mainly analyzed the upregulated circRNAs in the MM group. GO annotations were used to identify the top 10 biological process (BP), cellular component (CC) and molecular function (MF) enriched terms, which may reveal the main roles of upregulated circRNAs (Fig. 2A–C).

The results showed that activation of transmembrane receptor protein tyrosine kinase activity identified in the BP analysis was closely related to the resistance of myeloma cells. This activation can promote the survival and proliferation of myeloma cells and protect myeloma cells from apoptosis induced by bortezomib [34]. In addition, the CC term pericentral material was also closely related to MM. Excessive centrosome surrounding materials can cause abnormal mitosis and then affect myeloma cell apoptosis and cell cycle progression [35]. Moreover, histone methyltransferase activity (H3-K4 specific) identified in the MF analysis can also interfere with the occurrence of MM. Overexpression of the histone methyltransferase MMSET in t(4;14)+ MM patients is thought to be the driving factor in the pathogenesis of this myeloma subtype [36, 37]. KEGG analysis results showed that exo-circRNAs that were significantly upregulated in MM might regulate or participate in 38 signaling pathways. We selected the top 10 predicted signaling pathways based on the P value and selection counts (Fig. 2D). Among them, the "PI3K-Akt signaling pathway" plays an important role in myeloma cell proliferation, migration and apoptosis and can interfere with drug resistance in MM [38, 39]. The results showed that 3 of the top 10 KEGG pathways were related to cardiac disease. In addition, the descriptions "glutamate receptor pathway" and "calcium ion pathway" appeared repeatedly in GO and KEGG analyses. Previous studies have confirmed that calcium ions play an important role in regulating heart function [40]^.^ Abnormal levels of intracellular calcium ions can induce mitochondrial dysfunction and lead to heart failure [41, 42]. Glutamate receptors can promote apoptosis of ischemic human cardiomyocytes through the p38/MAPK pathway mediated by calcium influx [43]. Moreover, the ionotropic glutamate receptors NMDA and NMDAR1 can cause lipid peroxidation damage through intracellular calcium overload, which induces cardiomyocyte death [44]. The above results indicate that exo-circRNAs in the serum of MM patients are likely to participate in the process of MM-related myocardial damage through calcium ion pathways and glutamate receptor channels.

### PCR verification of exo-circRNA expression in MM patients

**Fig. 3:**
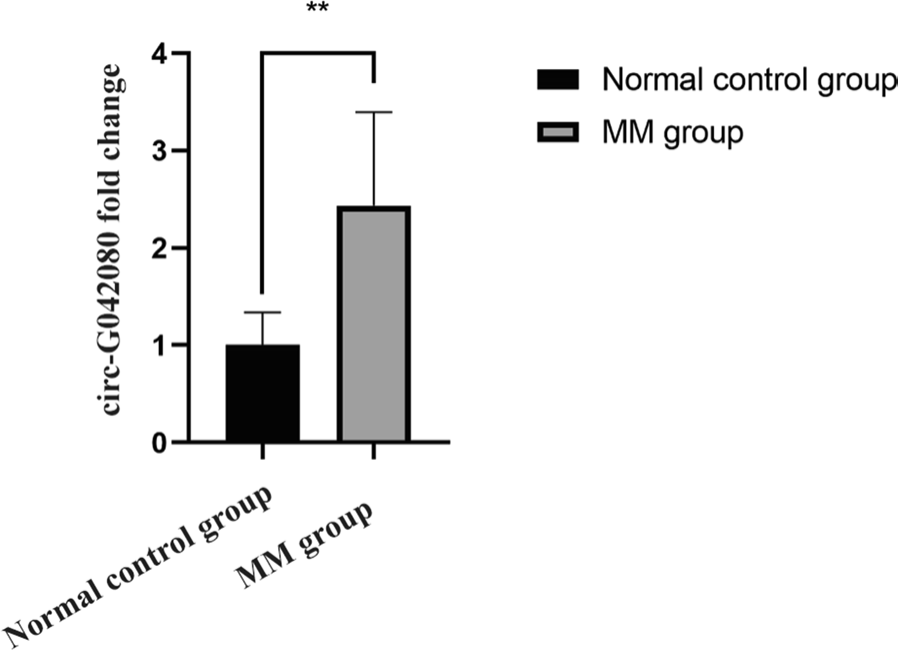
PCR validation of the abundant expression of exosomal circ-G042080 in the serum of MM patients. Every experiment was repeated in triplicate. ** P < 0.01 between the indicated pairs of groups

Based on the previous exo-circRNA sequencing results in MM patients and HCs and using the P value and fold changes, we selected 5 upregulated circRNAs and 8 downregulated circRNAs for PCR verification. The results were consistent with the high-throughput sequencing results. Through bioinformatics analysis, we found that the upregulated circRNA chr2: 2744264–2744443 + was closely associated with MM-related myocardial damage. Because it is an unknown circRNA, we named it circ-G042080 based on its gene location. The PCR results showed that the average relative circ-G042080 expression level in the MM group was more than two times that in the control group, which was consistent with the high-throughput sequencing results (Fig. 3). This result further confirmed the high circ-G042080 expression in the serum exosomes of MM patients.

### Construction of a circRNA-miRNA-mRNA interaction network and verification with a luciferase assay

**Fig. 4:**
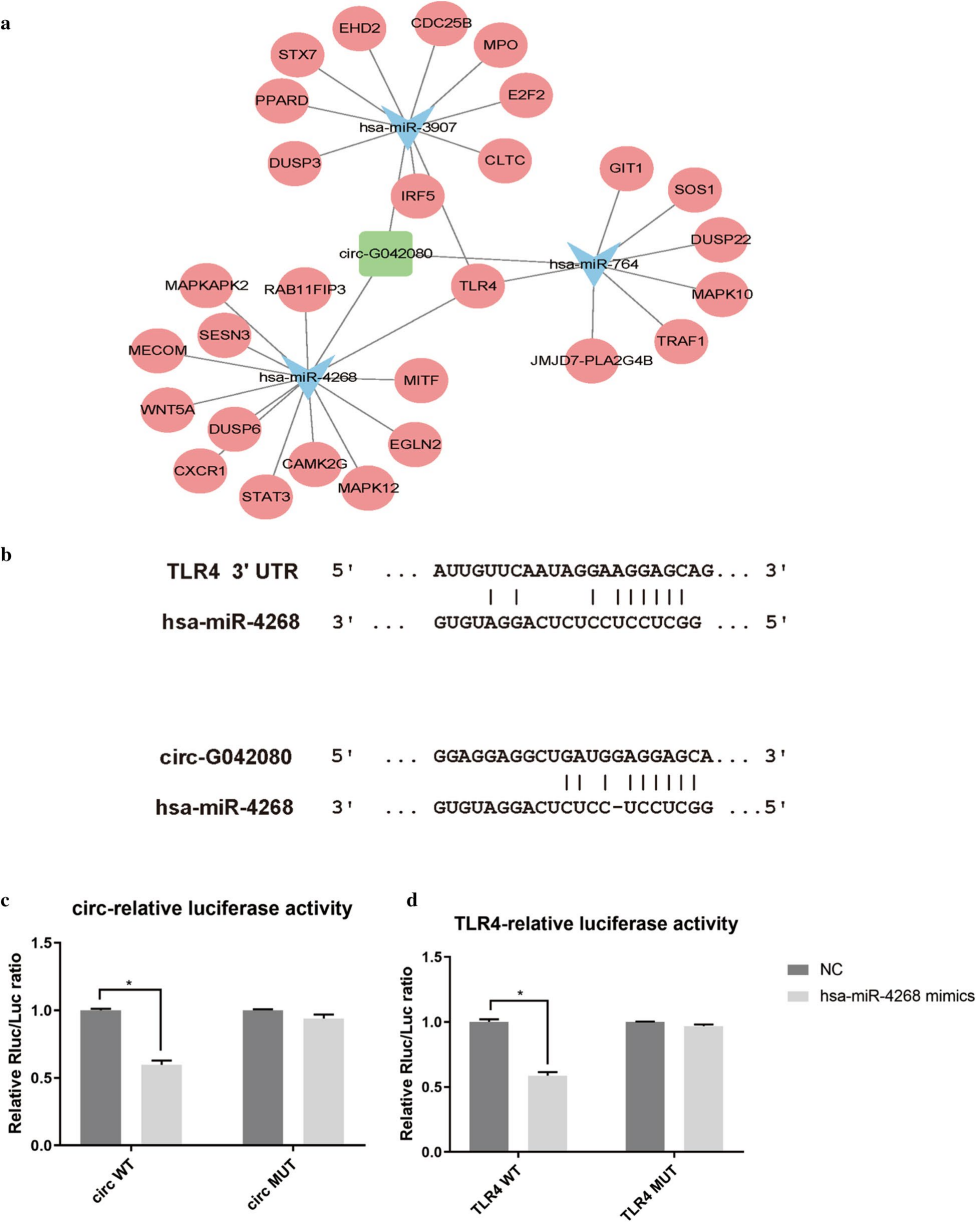
Construction of a circRNA-miRNA-mRNA interaction network and verification with a luciferase assay. **A** The circRNA-miRNA-mRNA network predicted by miRanda and TargetScan presented as a map constructed with Cytoscape software. **B** The binding sites between TLR4, hsa-miR-4268 and circ-G042080. **C** The luciferase assay results showed that hsa-miR-4268 mimics significantly reduced the luciferase activity in the circ-G042080 WT group compared with that in the HC group (P < 0.05) but had no effect in the circ-G042080 MUT group. **D** Luciferase activity results showed that hsa-miR-4268 mimics reduced the luciferase activity in the TLR4 WT group (P < 0.05) but had no effect on the luciferase activity in the TLR4 MUT group

circRNAs can affect miRNA function and miRNA expression by competing for miRNA binding sites. We predicted the downstream miRNAs and target genes of circ-G042080 using miRanda and TargetScan. The top 3 miRNAs with the strongest ability to interact with circ-G042080 were hsa-miR-4268, hsa-miR-764 and hsa-miR-3907, which we subjected to GO and KEGG analyses. Through KEGG analysis, among the top 10 enrichment pathways, we found that the Toll-like receptor signaling pathway was closely related to myocardial damage. Toll-like receptors can induce local heart inflammation and further aggravate myocardial damage [45]^.^ In addition, arrhythmogenic right ventricular cardiomyopathy (ARVC), hypertrophic cardiomyopathy (HCM) and dilated cardiomyopathy, which were also among the top 10 enriched pathways, are also closely related to myocardial damage. Cytoscape software was used to construct a ceRNA network diagram consisting of circ-G042080, miRNAs and downstream target genes. Figure 4A shows that 28 downstream target genes may bind to the top 3 miRNAs and that 4 of these target genes are involved in the Toll-like receptor signaling pathway. Among downstream mRNAs, TLR4, a member of the Toll-like receptor family, was regulated by all three miRNAs. Therefore, we chose to explore TLR4 as a possible downstream target gene in subsequent experiments. A dual-luciferase reporter assay was performed to determine direct binding between circ-G042080/TLR4 and miR-4268 based on their complementary sequences (Fig. 4B). The luciferase activity results showed that hsa-miR-4268 mimics decreased the luciferase activity in the circ-G042080 WT group (P < 0.05) and TLR4 WT group (P < 0.05) but had no effect on the luciferase activity in the circ-G042080 MUT group (P > 0.05) and TLR4 MUT group (P > 0.05) (Fig. 4C, D). The above results indicate that hsa-miR-4268 might target circ-G042080 and TLR4 expression, suggesting that circ-G042080 can activate the Toll-like receptor signaling pathway to induce MM-related myocardial damage by regulating downstream miRNAs and the TLR4 axis.

### Clinical relevance of the circ-G042080 level in MM-related myocardial damage

**Fig. 5:**
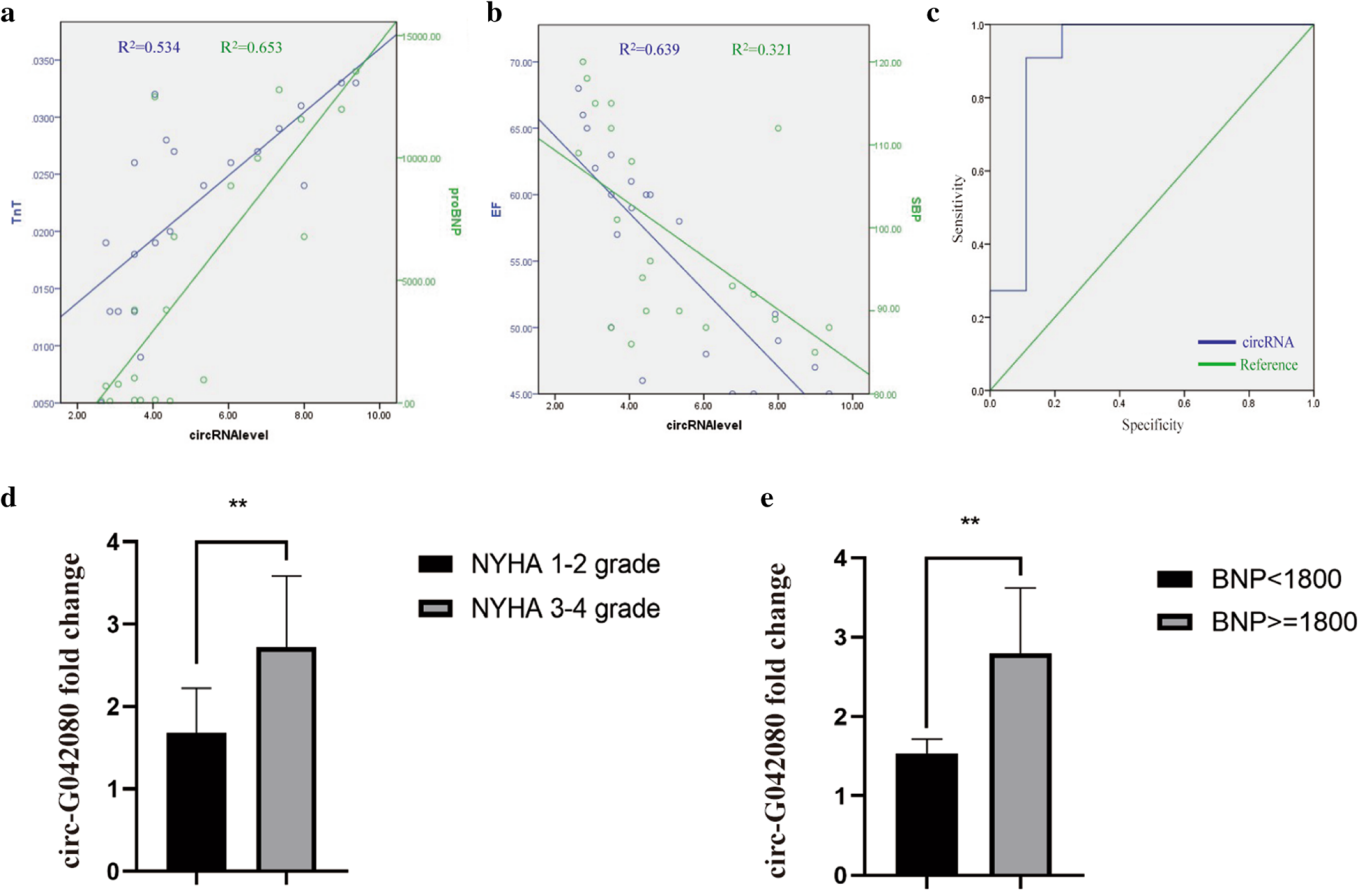
circ-G042080 is positively correlated with the clinical features of MM-related myocardial damage and is a potential prognostic indicator of MM-related myocardial damage. **A** circ-G042080 was found to be positively correlated with TnT (R^2^ = 0.534) and proBNP levels (R^2^ = 0.653). **B** circ-G042080 was negatively correlated with EF (R^2^ = 0.639) and SBP levels (R^2^ = 0.321). **C** ROC curve analysis showed that the level of circ-G042080 had high sensitivity and specificity for determination of the prognosis of patients with MM-related myocardial damage. The area under the curve (AUC) and optimal cutoff value of circ-G042080 were 0.09 and 2.18, respectively. **D** PCR verified the relationship between the serum exosome circ-G042080 level and the NYHA score in MM patients. **E** PCR verified the relationship between the serum exosomal circ-G042080 level and BNP level in MM patients

To further determine the clinical significance of circ-G042080 in MM-related myocardial damage, we analyzed the relationship between circRNA expression and the clinical characteristics of 20 MM patients. The results showed that circ-G042080 expression was positively correlated with TNT and proBNP levels (Fig. 5A). High circ-G042080 expression corresponded to a decrease in ventricular ejection fraction (EF) and systolic blood pressure (SPB) (Fig. 5B), which are markers of myocardial damage. Moreover, circ-G042080 expression was closely related to the clinical characteristics of MM-related myocardial damage, suggesting that circ-G042080 might be a reliable new biomarker of MM-related myocardial damage and a therapeutic target. In addition, the circ-G042080 level may also be an independent prognostic indicator of MM-related heart damage. The ROC curve showed that circ-G042080 levels had high sensitivity and specificity for predicting the prognosis of patients with MM-related myocardial damage, which suggests that circ-G042080 could be used as an effective clinical prognostic indicator. The area under the curve (AUC) of circ-G042080 was 0.909 (P = 0.05), corresponding to an optimal circ-G042080 cutoff value of 2.18 (Fig. 5C). Next, we further explored the relationship between clinical characteristics and survival rate using univariate and multivariate Cox regression analyses. Univariate Cox regression analysis showed that hemoglobin (HGB) < 100 g/L, D-S stage, NYHA stage, EF < 50%, NTproBNP ≥ 1800 and circ-G042080 ≥ 2.18 had significant negative effects on survival rate (P < 0.05). Multivariate Cox regression analysis showed that circ-G042080 ≥ 2.18 was an independent prognostic factor of death in patients with myeloma and cardiac failure (Table 2). Through PCR, we further explored the exosomal circRNA levels in patients with MM-related myocardial damage. The results showed that the circ-G042080 level in myeloma patients with NYHA grades 3–4 was significantly higher than that in patients with NYHA grades 1–2 (Fig. 5D). In addition, the circ-G042080 level in patients with BNP ≥ 1800 was significantly higher than that in patients with BNP < 1800 (Fig. 5E). These results indicate that the circ-G042080 level could be used as a reliable new biomarker of damage in myeloma and a prognostic factor.

**Table 2:** Univariate COX and multivariate COX regression analyses of potential prognostic factors related to MM-induced myocardial damage

Prognostic factor	Cumulative response rate of PN			
	Univariate Cox regression analysis		Multivariate Cox regression analysis	
	RR (95% CI)	*P* value	RR (95%CI)	*P* value
HGB ≥ 100 g/L	8.604 (1.038–71.335)	0.013	–	–
M% < 47.65%	1.794 (0.424–7.584)	0.419	–	–
D-S stage	0.398 (0.162–0.980)	0.072	–	–
NYHA grade	58.054 (0.248–13,597.283)	0.001	–	–
EF < 50%	0.86 (0.760–0.980)	0.005	–	–
NTproBNP ≥ 1800	58.054 (0.248–13,597.283)	0.001	–	–
circRNA < 2.18	107.551 (0.318–36,420.553)	0.000	107.551 (0.318–36,420.553)	0.000

*P < 0.1 was considered to be statistically significant in univariate Cox regression analysis, and P < 0.05 was considered to be statistically significant in multivariate Cox regression analysis

### Effects of exosomes from MM patients on H9C2 cells

**Fig. 6:**
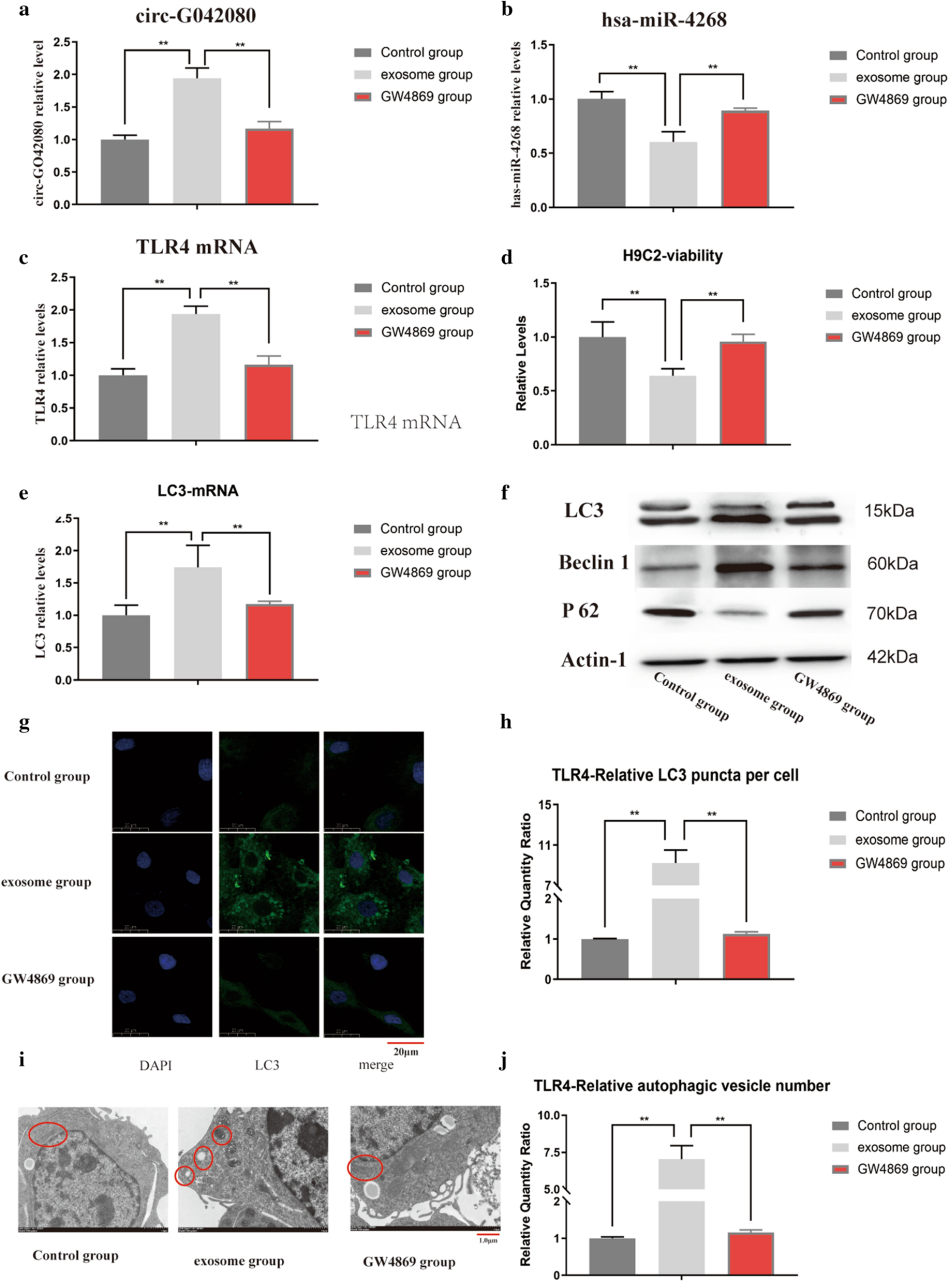
Effects of exosomes from MM patients on H9C2 cells. **A** The level of circ-G042080 was significantly increased in the exosome group (P < 0.01). After blocking the exosomes with GW4869, the level of circ-G042080 decreased. **B** The level of hsa-miR-4268 was significantly increased in the exosome group (P < 0.01) and then decreased after blocking exosomes with GW4869. **C**, **A** The level of TLR4 was significantly increased in the exosome group (P < 0.01) and then decreased after blocking exosomes with GW4869. **D** CCK-8 assay results showed that the proliferation rate of H9C2 cells in the exosome group was significantly reduced (P < 0.01) and that the cell proliferation rate increased after the exosomes were blocked with GW4869. **E** The level of LC3 was significantly increased in the exosome group (P < 0.01) and then decreased after blocking exosomes with GW4869. **F** WB results showed increased LC3-II/I and Beclin1 levels and a decreased P62 level in the exosome group compared with the control group. **G** Immunofluorescence results showing the LC3 expression level in H9C2 cells. **H** Compared with that in the control group, the LC3 expression level in the exosome group was significantly increased (P < 0.01). **I** Observation of the number of autophagic vesicles in H9C2 cells via electron microscopy. The red marks from left to right indicate mitochondria, autophagic vesicles, autophagic lysosomes, mitochondria, and mitochondria. **J** The results showed easily visible autophagic vacuoles in the exosome group, and the number was obviously greater than that in the control group

We designed a cell experiment to further explore the role of circ-G042080 overexpression in MM-related myocardial damage. H9C2 cells are often used to explore cardiomyocyte apoptosis, autophagy and toxic damage. In our experiment, exosomes from U266 cells were used to treat H9C2 cells to study the effect of circ-G042080 overexpression on cardiomyocytes. PCR results showed that the levels of circ-G042080 and TLR4 in the exosome group of H9C2 cells were significantly increased compared with levels in the control group (P < 0.01), while the hsa-miR-4268 level was significantly decreased (P < 0.01). However, after blocking exosomes with GW4869, the circ-G042080, hsa-miR-4268 and TLR4 levels were restored in H9C2 cells treated with exosomes from U266 cells (Fig. 6A–C). These results indicate that the circ-G042080/hsa-miR-4268/TLR4 ceRNA axis could be present in H9C2 cells incubated with exosomes. We further explored the effect of exosomes from U266 cells on the function of H9C2 cells. CCK-8 assay results indicated that the proliferation rate of H9C2 cells in the exosome group was significantly reduced compared with that in the control group and that exosomes overexpressing circ-G042080 had a significant antiproliferative effect (P < 0.01). After GW4869 was used to block exosomes, the proliferation rate of H9C2 cells affected by the exosomes of MM patients was restored (Fig. 6D). PCR experiments confirmed that LC3 levels in the exosome group were significantly increased compared with those in the control group and that this effect was reversed after the exosomes were blocked with GW4869 (P < 0.01) (Fig. 6E). WB results showed increased LC3 and Beclin1 levels and a decreased P62 level in the exosome group (Fig. 6F). The LC3 expression level was observed via immunofluorescence, and the results showed that the expression level in the exosome group was significantly higher than that in the control group (Fig. 6G, H). Additional File (1, 2) are the amplification curve and dissolution curve of TLR4 respectively.

We further observed autophagic vesicles through electron microscopy, and the results showed that the number of autophagic vesicles in the exosome group was significantly greater than that in the control group (Fig. 6 I, J). The above results suggest that circ-G042080 in exosomes activated TLR4 in H9C2 cells through the ceRNA mechanism, thereby inducing autophagic death in cardiomyocytes.

## Discussion

Exosomes are membrane-like structures that carry signaling molecules and have received extensive attention as important mediators of intercellular communication. Previous studies have confirmed that by delivering abundant biologically active molecules (including miRNAs, growth factors, and signaling molecules) [46] exosomes can participate in the growth and drug resistance of several tumors, such as gynecologic cancers [47, 48] and malignant glioma [49].In addition, exosomes have been verified to mediate bidirectional transfer of proteins, lipids and nucleic acids between the bone marrow microenvironment and MM and promote MM development by promoting angiogenesis, osteolysis and drug resistance [50]. Exosomes have broad prospects as biomarkers for early prediction of MM disease progression and drug resistance [51].

Amyloidosis is a serious complication of MM and is primarily caused by structural changes and extracellular deposition of monoclonal immunoglobulins [52]. In the clinic, cardiac involvement is the second most common manifestation after kidney involvement. Heart involvement usually has no characteristic clinical symptoms at the beginning. As the disease progresses, restrictive cardiomyopathy, heart failure, and abnormal rhythms will appear and then develop into heart failure [53], which seriously affects patient survival and quality of life. At present, pathological biopsy is the only way to diagnose myocardial amyloidosis in MM. Therefore, we urgently need a new early target index to diagnose and treat myocardial amyloidosis and related heart damage in MM. circRNAs are noncoding RNAs that are widely present in human body fluids. Previously, circRNAs were though to result from random errors that occurred during transcription. As evidence accumulated, circRNAs were shown to serve as potentially important prognostic and diagnostic factors in cancer due to their high stability and specific expression [54, 55]. A large amount of evidence has also shown that circRNAs are key regulators of MM [56]. In addition, circRNAs are closely related to heart failure and can predict heart failure development and aid in risk stratification [57]. A previous study by our team found that exo-circRNAs have excellent diagnostic and therapeutic potential for MM-related peripheral neuropathy (PN) [58]. Unfortunately, research on the relationship between MM-related myocardial damage and exosomes is still relatively rare.

To further explore the mechanism of action of MM-derived exosomes in MM and MM-related myocardial damage, we extracted exosomes from the serum of 6 MM patients and 5 healthy individuals and studied the expression levels of exo-circRNAs (Fig. 1A–C). The data showed that 2052 circRNAs were differentially expressed between MM patients and HCs (Fig. 1D, E). We focused on the characteristics of exo-circRNAs that were significantly upregulated in MM. GO and KEGG analyses indicated the role of these circRNAs in the pathogenesis of MM progression and myocardial damage. Among the enriched pathways, the "PI3K-Akt signaling pathway" plays an important role in myeloma cell proliferation, migration and apoptosis [38, 39] (Fig. 2). In addition, bioinformatics analysis predicted that circ-G042080 might regulate a downstream miRNA/TLR4 axis (Fig. 4). At present, TLR4 has been identified as a crucial regulator in cardiovascular disease. Studies have found that TLR4 activation in rats promotes cardiac inflammation, mitochondrial dysfunction, apoptosis and fibrosis [59, 60]. In addition, the "glutamate receptor pathway" and "calcium ion pathway" were often identified in GO and KEGG analyses. Previous studies have confirmed that calcium ions play an important role in regulation of heart function [40], and glutamate receptors can promote the death of ischemic human cardiomyocytes through the p38/MAPK pathway mediated by calcium influx [43] (Fig. 2).

These findings indicate that exo-circRNAs in the serum of MM patients are likely to participate in the development of MM-related myocardial damage through their effect on calcium channels and glutamate receptor channels. This suggests that circ-G042080 might regulate downstream miRNAs and TLR4 to induce heart damage through a molecular sponge mechanism.

We further explored the relationship between circ-G042080 and the clinical characteristics of MM patients. The results showed that circ-G042080 was closely related to the clinical features of heart damage (Fig. 5A, B), which suggested that circ-G042080 is a promising marker of MM-related heart damage. The ROC curve suggested that circ-G042080 could be used as an effective clinical prognostic indicator (Fig. 5C). Univariate and multivariate Cox analyses further confirmed that circ-G042080 was an independent prognostic factor for the death of patients with myeloma and heart failure (Table 2). Subsequently, we treated H9C2 cells with exosomes derived from MM patients and found that the levels of circ-G042080 and TLR4 in these cells increased and that the level of hsa-miR-4268 decreased. These effects were reversed after exosomes were blocked with GW4869 (Fig. 6A–C). The sphingomyelinase inhibitor GW4869 is a recognized exosome inhibitor and is thought to inhibit exosome secretion. We further explored the effect of exosomes on the function of H9C2 cells. CCK-8 assays revealed that the proliferation rate of H9C2 cells treated with exosomes derived from MM patients was significantly reduced and that the proliferation rate increased after the exosomes were blocked with GW4869. LC3, Beclin1, and P62 are all autophagy-related markers. LC3 is a commonly used autophagy marker. LC3-I is converted into LC3-II when autophagy is initiated. LC3-II, which is the structural protein of the autophagosome, can be adsorbed on the autophagosome membrane. The ratio of LC3-II/I can reflect the autophagy level. In addition, the autophagy-specific gene Beclin1 can participate in the occurrence of a variety of tumors by regulating the strength of autophagy [61, 62]. Moreover, P62 can interact with LC3 to target it for degradation in autophagic lysosomes [63].

Autophagy is a highly conserved catabolic process used to remove abnormal cytoplasmic components, including protein aggregates and damaged organelles [64]. Inflammatory factors participate in the inflammatory response mainly through TLRs. There are many subtypes of TLRs, of which TLR4 is the most highly expressed in the heart [65]. In recent years, studies have found that there is a close relationship between autophagy and inflammation, and autophagy has been shown to be an important component of the innate immune response [66]. Studies have confirmed that TLR4 can be bound by endogenous ligands to recognize cell surface antigens and then activate autophagy [67]. Our experiments demonstrated that the autophagy level was significantly increased in H9C2 cells cocultured with MM patient exosomes and that GW4869 blocked this effect (Fig. 6E–J). The above results suggested that circ-G042080 in exosomes could activate H9C2 cells to overexpress TLR4 through the ceRNA mechanism and then induce autophagic death of cardiomyocytes. Overall, our research showed that exo-circRNAs have broad application potential in early diagnosis of MM myocardial amyloidosis and in targeted therapy for MM-related myocardial damage. However, in further explorations of the usability of exo-circRNAs in MM-related myocardial damage, the patient sample size must be increased. In addition, we only employed in vitro experiments to explore circRNAs and did not use in vitro models to conduct further studies, which is the plan for our future work.

## Conclusion

Our study found that exo-circRNAs are abundant in the serum of MM patients. Bioinformatics analysis showed that upregulation of circ-G042080 expression in exosomes might interfere with MM-related myocardial damage by regulating miRNA and the TLR4 axis, which can influence autophagy in the myocardium. Moreover, the circ-G042080 expression level was positively correlated with the clinical features of heart damage in MM patients and thus could be used as an independent prognostic indicator of myocardial damage. Cell experiments further confirmed that the circ-G042080/hsa-miR-4268/TLR4 ceRNA axis might exist in H9C2 cells incubated with exosomes and can cause an increase in the autophagy level in cardiomyocytes. In summary, our results indicate that serum exo-circRNAs have the potential to serve as new biomarkers of MM-related myocardial damage and might be therapeutic targets. However, the mechanism remains to be further explored.

## Supplementary Information

**Additional file1:** Amplification curve of TLR4.

**Additional file2:** Dissolution curve of TLR4.

## Data Availability

The datasets generated and/or analyzed in the current study are not publicly available because the data are in the confidential stage but are available from the corresponding author upon reasonable request.

## Abbreviations

MM: Multiple myeloma
HCs: Healthy controls
circRNAs: Circular RNAs
exo-circRNAs: Exosomal circular RNAs
ceRNAs: Competitive endogenous RNAs
EVs: Extracellular vesicles
TEM: Transmission electron microscopy
BP: Biological process
CC: Cellular component
MF: Molecular function
ARVC: Arrhythmogenic right ventricular cardiomyopathy
HCM: Hypertrophic cardiomyopathy
EF: Ejection fraction
SBP: Systolic blood pressure
AUC: Area under the curve
HGB: Hemoglobin
PN: Peripheral neuropathy
TLR4: Toll-like receptor 4
qRT-PCR: Real-time qPCR
CCK-8: Cell counting kit-8
SD: Standard deviation
ROC: Receiver operating characteristic
rRNA: Ribosomal RNA
DMED: Dulbecco’s modified Eagle’s medium
RPMI: Roswell Park Memorial Institute
FC: Fold change
ARVC: Arrhythmogenic right ventricular cardiomyopathy
HCM: Hypertrophic cardiomyopathy
SDS-PAGE: Sodium dodecyl sulphate–polyacrylamide gel electrophoresis
PVDF: Polyvinylidene fluoride
BCA: Bicinchoninic acid

## References

[1] Mateos MV, San Miguel JF (2017). Management of multiple myeloma in the newly diagnosed patient. Hematology Am Soc Hematol Educ Program.

[2] Mirzaei H, Bagheri H, Ghasemi F (2021). Anti-Cancer Activity of Curcumin on Multiple Myeloma. Anticancer Agents Med Chem.

[3] Plummer C, Driessen C, Szabo Z (2019). Management of cardiovascular risk in patients with multiple myeloma. Blood Cancer J.

[4] Sattar Y, Ruiz Maya T, Zafrullah F (2018). Diagnosis and management of a cardiac amyloidosis case mimicking hypertrophic cardiomyopathy. Cureus..

[5] Li Y, Zheng Q, Bao C (2015). Circular RNA is enriched and stable in exosomes: a promising biomarker for cancer diagnosis. Cell Res.

[6] Ng WL, Mohd Mohidin TB, Shukla K (2018). Functional role of circular RNAs in cancer development and progression. RNA Biol.

[7] Zhang Z, Yang T, Xiao J (2018). Circular RNAs: promising biomarkers for human diseases. EBioMedicine.

[8] Memczak S, Jens M, Elefsinioti A (2013). Circular RNAs are a large class of animal RNAs with regulatory potency. Nature.

[9] Razavi ZS, Tajiknia V, Majidi S (2021). Gynecologic cancers and non-coding RNAs: epigenetic regulators with emerging roles. Crit Rev Oncol Hematol.

[10] Shabaninejad Z, Vafadar A, Movahedpour A (2019). Circular RNAs in cancer: new insights into functions and implications in ovarian cancer. J Ovarian Res.

[11] Song C, Li D, Liu H (2019). The competing endogenous circular RNA ADAMTS14 suppressed hepatocellular carcinoma progression through regulating microRNA-572/regulator of calcineurin 1. J Cell Physiol.

[12] Liu H, Bi J, Dong W (2018). Invasion-related circular RNA circFNDC3B inhibits bladder cancer progression through the miR-1178-3p/G3BP2/SRC/FAK axis. Mol Cancer.

[13] Liu H, Liu Y, Bian Z (2018). Circular RNA YAP1 inhibits the proliferation and invasion of gastric cancer cells by regulating the miR-367-5p/p27 (Kip1) axis. Mol Cancer.

[14] Wang H, Xiao Y, Wu L (2018). Comprehensive circular RNA profiling reveals the regulatory role of the circRNA-000911/miR-449a pathway in breast carcinogenesis. Int J Oncol.

[15] Li X, Diao H (2019). Circular RNA circ_0001946 acts as a competing endogenous RNA to inhibit glioblastoma progression by modulating miR-671-5p and CDR1. J Cell Physiol.

[16] Shang J, Chen WM, Wang ZH (2018). CircPAN3 mediates drug resistance in acute myeloid leukemia through the miR-153-5p/miR-183-5p-XIAP axis. Exp Hematol.

[17] Li S, Ma Y, Tan Y (2018). Profiling and functional analysis of circular RNAs in acute promyelocytic leukemia and their dynamic regulation during all-trans retinoic acid treatment. Cell Death Dis.

[18] Dahl M, Daugaard I, Andersen MS (2018). Enzyme-free digital counting of endogenous circular RNA molecules in B-cell malignancies. Lab Invest.

[19] Abbaszadeh-Goudarzi K, Radbakhsh S, Pourhanifeh MH (2020). Circular RNA and diabetes: epigenetic regulator with diagnostic role. Curr Mol Med.

[20] Pourhanifeh MH, Mahjoubin-Tehran M, Shafiee A (2020). MicroRNAs and exosomes: Small molecules with big actions in multiple myeloma pathogenesis. IUBMB Life.

[21] Feng Y, Zhang L, Wu J (2019). CircRNA circ_0000190 inhibits the progression of multiple myeloma through modulating miR-767-5p/MAPK4 pathway. J Exp Clin Cancer Res.

[22] Zhou F, Wang D, Wei W (2020). Comprehensive profiling of circular RNA expressions reveals potential diagnostic and prognostic biomarkers in multiple myeloma. BMC Cancer.

[23] Fan S, Hu K, Zhang D (2020). Interference of circRNA HIPK3 alleviates cardiac dysfunction in lipopolysaccharide-induced mice models and apoptosis in H9C2 cardiomyocytes. Ann Transl Med.

[24] Wang Y, Zhao R, Liu W (2019). Exosomal circHIPK3 released from hypoxia-pretreated cardiomyocytes regulates oxidative damage in cardiac microvascular endothelial cells via the miR-29a/IGF-1 pathway. Oxid Med Cell Longev.

[25] Raposo G, Stoorvogel W (2013). Extracellular vesicles: exosomes, microvesicles, and friends. J Cell Biol.

[26] Michael A, Bajracharya SD, Yuen PS (2010). Exosomes from human saliva as a source of microRNA biomarkers. Oral Dis.

[27] Zijlstra A, Di Vizio D (2018). Size matters in nanoscale communication. Nat Cell Biol.

[28] Lee YS, Kim SY, Ko E (2017). Exosomes derived from palmitic acid-treated hepatocytes induce fibrotic activation of hepatic stellate cells. Sci Rep.

[29] Liu H, Li B (2018). The functional role of exosome in hepatocellular carcinoma. J Cancer Res Clin Oncol.

[30] Faict S, Muller J, De Veirman K (2018). Exosomes play a role in multiple myeloma bone disease and tumor development by targeting osteoclasts and osteoblasts. Blood Cancer J.

[31] Li M, Xia B, Wang Y (2019). Potential therapeutic roles of exosomes in multiple myeloma: a systematic review. J Cancer.

[32] Raimondo S, Saieva L, Vicario E (2019). Multiple myeloma-derived exosomes are enriched of amphiregulin (AREG) and activate the epidermal growth factor pathway in the bone microenvironment leading to osteoclastogenesis. J Hematol Oncol.

[33] Roccaro AM, Sacco A, Maiso P (2013). BM mesenchymal stromal cell-derived exosomes facilitate multiple myeloma progression. J Clin Invest.

[34] Waizenegger JS, Ben-Batalla I, Weinhold N (2015). Role of Growth arrest-specific gene 6-Mer axis in multiple myeloma. Leukemia.

[35] Maxwell CA, Keats JJ, Belch AR (2005). Receptor for hyaluronan-mediated motility correlates with centrosome abnormalities in multiple myeloma and maintains mitotic integrity. Cancer Res.

[36] Popovic R, Martinez-Garcia E, Giannopoulou EG (2014). Histone methyltransferase MMSET/NSD2 alters EZH2 binding and reprograms the myeloma epigenome through global and focal changes in H3K36 and H3K27 methylation. PLoS Genet.

[37] Ren Z, Ahn JH, Liu H (2019). PHF19 promotes multiple myeloma tumorigenicity through PRC2 activation and broad H3K27me3 domain formation. Blood.

[38] Jiang Y, Chang H, Chen G (2018). Effects of microRNA-20a on the proliferation, migration and apoptosis of multiple myeloma via the PTEN/PI3K/AKT signaling pathway. Oncol Lett.

[39] Wang L, Lin N, Li Y (2019). The PI3K/AKT signaling pathway regulates ABCG2 expression and confers resistance to chemotherapy in human multiple myeloma. Oncol Rep.

[40] Coetzee WA (1988). Channel-mediated calcium current in the heart. Cardiovasc Drugs Ther.

[41] Marx SO, Reiken S, Hisamatsu Y, Jayaraman T (2000). PKA phosphorylation dissociates FKBP12.6 from the calcium release channel (ryanodine receptor): defective regulation in failing hearts. Cell.

[42] Santulli G, Xie W, Reiken SR (2015). Mitochondrial calcium overload is a key determinant in heart failure. Proc Natl Acad Sci USA.

[43] Liu ZY, Zhong QW, Tian CN (2019). NMDA receptor-driven calcium influx promotes ischemic human cardiomyocyte apoptosis through a p38 MAPK-mediated mechanism. J Cell Biochem.

[44] Sun Y, Sun B, He R (2017). Effect of the changes of NMDA receptor in hypothalamic paraventricular nucleus on cardiac function and sympathetic nervous activity in rats with heart failure. Biochem Biophys Res Commun.

[45] Han Y, Liao X, Gao Z (2016). Cardiac troponin I exacerbates myocardial ischaemia/reperfusion injury by inducing the adhesion of monocytes to vascular endothelial cells via a TLR4/NF-κB-dependent pathway. Clin Sci.

[46] Gilligan KE, Dwyer RM (2017). Engineering exosomes for cancer therapy. Int J Mol Sci.

[47] Nahand JS, Vandchali NR, Darabi H (2020). Exosomal microRNAs: novel players in cervical cancer. Epigenomics.

[48] Hashemipour M, Boroumand H, Mollazadeh S (2021). Exosomal microRNAs and exosomal long non-coding RNAs in gynecologic cancers. Gynecol Oncol.

[49] Ghaemmaghami AB, Mahjoubin-Tehran M, Movahedpour A (2020). Role of exosomes in malignant glioma: microRNAs and proteins in pathogenesis and diagnosis. Cell Commun Signal.

[50] Chen T, Moscvin M, Bianchi G (2020). Exosomes in the pathogenesis and treatment of multiple myeloma in the context of the bone marrow microenvironment. Front Oncol.

[51] Moloudizargari M, Abdollahi M, Asghari MH (2019). The emerging role of exosomes in multiple myeloma. Blood Rev.

[52] Desport E, Bridoux F, Sirac C (2012). Al amyloidosis. Orphanet J Rare Dis.

[53] Zhang Q, Qiao Y, Yan D (2020). Myocardial amyloidosis following multiple myeloma in a 38-year-old female patient: a case report. Open Med.

[54] Perez de Acha O, Rossi M, Gorospe M (2020). Circular RNAs in Blood Malignancies. Front Mol Biosci.

[55] Nahand JS, Jamshidi S, Hamblin MR (2020). Circular RNAs: new epigenetic signatures in viral infections. Front Microbiol.

[56] Song Y, Hu N, Song X (2020). Hsa_Circ_0007841 enhances multiple myeloma chemotherapy resistance through upregulating ABCG2. Technol Cancer Res Treat.

[57] Carrara M, Fuschi P, Ivan C (2018). Circular RNAs: Methodological challenges and perspectives in cardiovascular diseases. J Cell Mol Med.

[58] Zhang Y, Pisano M, Li N (2021). Exosomal circRNA as a novel potential therapeutic target for multiple myeloma-related peripheral neuropathy. Cell Signal.

[59] Xiao Z, Kong B, Yang H (2020). Key player in cardiac hypertrophy, emphasizing the role of toll-like receptor 4. Front Cardiovasc Med.

[60] Katare PB, Nizami HL, Paramesha B (2020). Activation of toll like receptor 4 (TLR4) promotes cardiomyocyte apoptosis through SIRT2 dependent p53 deacetylation. Sci Rep.

[61] Brier LW, Ge L, Stjepanovic G (2019). Regulation of LC3 lipidation by the autophagy-specific class III phosphatidylinositol-3 kinase complex. Mol Biol Cell.

[62] Xu HD, Qin ZH (2019). Beclin1, Bcl-2 and autophagy. Adv Exp Med Bol.

[63] Nam T, Han JH, Devkota S (2017). Emerging paradigm of crosstalk between autophagy and the ubiquitin-proteasome system. Mol Cells.

[64] Qin C, Liu Q, Hu ZW (2018). Microglial TLR4-dependent autophagy induces ischemic white matter damage via STAT1/6 pathway. Theranostics.

[65] Oyama J, Blais C, Liu X (2004). Reduced myocardial ischemia-reperfusion injury in toll-like receptor 4-deficient mice. Circulation.

[66] Arroba AI, Rodríguez-de la Rosa L, Murillo-Cuesta S (2016). Autophagy resolves early retinal inflammation in Igf1-deficient mice. Dis Model Mech.

[67] Yang X, Sun C, Wang L (2019). New insight into isolation, identification techniques and medical applications of exosomes. J Control Release.

