# Supplementary figures and images for: Exosomal circRNA as a novel potential therapeutic target for multiple myeloma-related myocardial damage
Source: Cancer Cell Int. 2021 Jun 13;21:311. doi: 10.1186/s12935-021-02011-w (PMC8201884; doi:10.1186/s12935-021-02011-w)

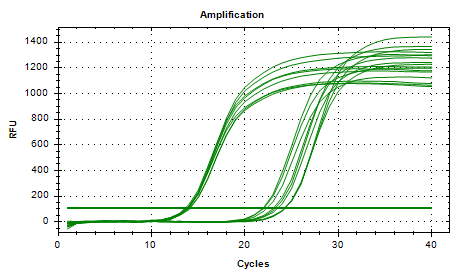

Supplement: Supplementary file 1 — Additional file1: Amplification curve of TLR4. [file 12935_2021_2011_MOESM1_ESM.png]

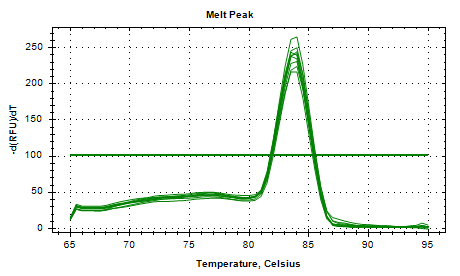

Supplement: Supplementary file 2 — Additional file2: Dissolution curve of TLR4. [file 12935_2021_2011_MOESM2_ESM.png]
